# The impact of obesity and overweight on medical expenditures and disease incidence in Korea from 2002 to 2013

**DOI:** 10.1371/journal.pone.0197057

**Published:** 2018-05-10

**Authors:** Hyun Jin Song, Jinseub Hwang, Seonmi Pi, Sena Ahn, Yoonseok Heo, Susan Park, Jin-Won Kwon

**Affiliations:** 1 College of Pharmacy and Research Institute of Pharmaceutical Sciences, Kyungpook National University, Daegu, South Korea; 2 College of Pharmacy, University of Florida, Gainesville, Florida, United States of America; 3 Major in Statistics and Data Science, Daegu University, Gyeongbuk, South Korea; 4 Department of Surgery, Inha University College of Medicine, Incheon, South Korea; TNO, NETHERLANDS

## Abstract

**Objectives:**

Few studies have assessed the long-term medical costs and incidence of obesity and overweight in Asia. We evaluated the impact of body mass index (BMI) on medical expenditures and disease incidence and prevalence over more than 10 years in South Korea.

**Methods:**

Using 2002–2013 data from the Korean National Claims Database, we analysed two population sets (initial BMI in 2002–2003; consistent BMI in 2002–2003 and 2012–2013). Obesity was defined by Asian BMI criteria. Incremental medical expenditures or Charlson Comorbidity Index (CCI) ratios for obese compared to normal weight individuals were calculated. Medical expenditure over 11 years was estimated by BMI using a generalised linear model. Individual obesity-related disease incidence was determined and adjusted hazard ratios were calculated.

**Results:**

Data for 496,469 and 214,477 individuals were included in the entire and consistent BMI level populations, respectively. Average CCI score change in normal weight and the obesity III (BMI 35–59.99 kg/m^2^) group over 11 years were 0.94 and 1.56, respectively in the entire population, and incremental ratio in the obesity III group was 66.0% compared to the normal weight group. In consistent BMI level population, incremental ratio (92.1%) for obesity III was higher than entire population. Medical costs in the obesity III groups versus the normal weight group in the entire and consistent BMI level populations increased by 38.4% and 77.1%, respectively. Over 11 years, individuals with BMI ≥30 kg/m^2^ in the entire and consistent BMI level populations had post-adjustment medical expenditures of 1.13–1.20 and 1.21–1.40 times the normal weight group, respectively. Incidence rate and adjusted hazard ratio of obesity-related disease increased in the obesity groups compared to the normal weight group.

**Conclusions:**

Our findings emphasize the importance of the effective and sustainable obesity management strategies, considering the dramatic increase in obesity (BMI ≥30 kg/m^2^) in South Korea.

## Introduction

Since the World Health Organization (WHO) classified obesity as a disease, various measures to decrease obesity have been considered worldwide [1]. However, the prevalence of obesity has not decreased, and it remains an urgent public health agenda in both developed and developing countries. From 1980 to 2013, the prevalence of obesity or overweight increased worldwide by 27.5% and 47.1% in adults and children, respectively [2]. The prevalence of obesity differs by sex, age, and socioeconomic status [3]. In particular, a rapid increase in the prevalence of obesity among younger populations and those of lower socioeconomic status has been observed.

Obesity has been a major public health issue both in itself and as a result of its association with the development of several chronic comorbidities. Obesity-related diseases are not limited to cardiovascular diseases. There is abundant evidence of the association between obesity and cancer, respiratory, and neurodegenerative diseases [4–5].

Many countries are faced with dramatic increases in medical expenditures owing to the rising prevalence of chronic diseases. Accordingly, these countries seek to decrease medical costs and increase healthy life expectancies. The increased prevalence of chronic diseases may be the result of the increased number of elderly individuals. This factor, combined with the increasing prevalence of obesity, may be responsible for a substantial proportion of the disease burden [6]. Developed countries track both the social and medical costs of obesity and prioritise obesity prevention in health agendas in order to decrease medical expenditures.

In the US, medical costs related to obesity comprise more than 20% of the annual national healthcare expenditure [7]. The direct medical costs of obese adults are more than 42% higher than those of adults of normal weight [8] and more than 81% higher in severely obese adults (body mass index [BMI] >40 kg/m^2^) [9]. The seriousness of obesity-related comorbidities or costs has been reported in developed countries as well as the US [10–12].

Asian individuals experience obesity-related diseases at lower BMIs compared to those in Western countries [13]. However, few studies have assessed medical costs and disease incidence by BMI in Asian countries, including South Korea. Furthermore, there have been few studies regarding the long-term impact of obesity and overweight. Thus, the aim of this study was to evaluate the impact of BMI on medical expenditures and disease incidence and prevalence over more than 10 years in South Korea using a national longitudinal database from 2002–2013.

## Materials and methods

### Data sources

We used data from the Korean National Health Insurance Service-Health Screening Cohort (NHIS-HEALS) database from 2002 to 2013 [14–15]. Raw data can be provided to the researchers upon reasonable request and with the permission of the Korean National Health Insurance Service (NHIS) Institutional Data Access (http://nhiss.nhis.or.kr). We did not have any special access privileges.

The NHIS-HEALS included all NHI claimed and screened medical information for 514,866 health screening participants who comprised a 10% random sample of all health screening participants in 2002 and 2003. The participation rate of the general health screening among the eligible population in 2014 was 74.8% [15]. Although we could not rule out potential selection bias in participation in the health screening, previous studies using Korean National Health and Nutrition Examination Survey (KNHANES) data showed obesity prevalence similar to our results [16–17]. For the direct comparison, we presented the obesity prevalence in our database and analysed data for subjects aged 40–79 years from KNHANES II (2001) and III (2005) (S1 Table). Our obesity prevalence was within the confidence interval of obesity prevalence using KNHANES II data.

The NHIS-HEALS database contains four databases on insurance eligibility, medical treatments, medical care institutions, and general health examinations. The database on insurance eligibility includes information related to beneficiary qualifications (e.g. income level, death record). Individuals 40 years of age or older are required undergoing general health examinations every two years; these data were captured in the general health examination database. The medical treatment database included disease diagnosis (International Codes of Disease 10^th^ Edition Clinical Modification, ICD-10-CM), medical procedures, prescription drugs, and medical expenditures covered under the NHI. The Institutional Review Board of Kyungpook National University (KNU 2016–0077) approved this study. Informed consent was not required because the patient records from the NHIS-HEALS were anonymised and de-identified.

### Participants

Among 514,350 health screening participants, individuals with cancer (n = 17,103, 3.3%) or women who were pregnant (n = 754, 0.1%) were excluded because they might have a disproportionate influence on weight. In addition, individuals with extreme BMI values, such as more than 60 kg/m^2^ (n = 24, 0.005%), were excluded, as in another study in South Korea [18]. In our database, there were very few individuals with BMI ≥35 kg/ m^2^ (n = 870, 0.2%). Therefore, obese individuals with BMI ≥60 kg/ m^2^ constituted a very small portion, and there was a high probability of wrong coding data.

For this analysis, we used two populations. First, we selected individuals with weight and height measurements performed in 2002–2003 from the general health examination data of the NHIS-HEALS database; the first population set was the entire population. Second, individuals whose BMI remained in the same category at baseline (2002–2003) and at the observation end date (2012–2013) were selected in order to investigate the weight effects in detail; this group constituted the consistent BMI level population set. The number of two populations set was specified in the S1 Fig. In the entire population set, some individuals died before 2012–2013, but all individuals included in consistent BMI level population set lived for the entire 11-year follow-up period. The analysis of two populations was considered because we could not fully capture BMI changes throughout the follow-up periods and many individuals had only baseline BMI measurements. In the entire population set, the long-term effects of baseline BMI on clinical outcomes (i.e., disease prevalence, incidence, and medical expenditures) can be observed, regardless of weight change effects. Through analysis of the population with consistent BMI levels, we can further investigate the impact on clinical outcome by BMI level consistency.

We observed the 11-year changes in Charlson Comorbidity Index (CCI) scores and medical expenditure. Because general health examinations were conducted every two years, the follow-up BMIs for individuals with first measurements in 2002 and 2003 were measured in 2012 and 2013, respectively.

### Variables

#### BMI categories

BMI was determined from weight and height measurements as weight (kg) divided by the square of the height (m^2^). According to Asian criteria, BMI was defined as underweight (<18.5 kg/m^2^), normal weight (18.5–22.99 kg/m^2^), overweight (23–24.99 kg/m^2^), obesity I (25–29.99 kg/m^2^), obesity II (30–34.99 kg/m^2^), and obesity III (35–59.99 kg/m^2^) [19–20]. There were some differences in the definition of obesity according to BMI level between Asian and Western individuals. Underweight was the same according to both criteria; however, normal weight and overweight according to Asian criteria are considered normal weight by Western criteria. The obesity I, obesity II, and obesity III groups by Asian criteria were defined as overweight, obesity I, and obesity II groups by Western criteria.

#### Income level

Participant income levels were grouped using insurance type and level of insurance at baseline. Beneficiaries were divided into district subscribers, employee subscribers, and medical aids. In the case of district and employee subscribers, 10 levels in each group are available based on insurance amount. Higher scores represent a higher income level. We divided district and employee subscribers into three groups with similar proportions.

#### Comorbidities

Comorbidities were investigated using CCI scores. The CCI was originally developed to evaluate the mortality risk of concurrent diseases. The diseases included in the CCI are congestive heart failure (CHF), myocardial infarction (MI), cerebrovascular disease, peripheral vascular disease, connective tissue disease, chronic lung disease, ulcer, chronic liver disease, severe liver disease, dementia, diabetes, hemiplegia, moderate or severe kidney disease, tumour, leukaemia, lymphoma, moderate or metastatic solid tumour, and acquired immunodeficiency syndrome (AIDS). Each disease receives a score (i.e., diseases with higher mortality risks have higher scores) and the scores are summed to show the mortality risk of an individual. Quan et al. proposed methods to calculate CCI scores using ICD codes in administrative data [21].

Hypertension and depression, diseases related to obesity that are not included in the CCI, were also considered as comorbidities. Hypertension and depression were defined using ICD-10 codes I10-15 and F32-33, respectively.

### Outcomes

#### Changes in CCI score and medical expenditures between 2002–2003 and 2012–2013

We investigated the changes in CCI scores and medical expenditures by BMI category for 11 years by comparing the average CCI score and medical expenditures between participants at baseline and at the end of the observation period among survivors. For example, if the BMI of a participant was available in 2002, the average CCI scores and medical expenditures in 2002 and 2012 were assessed.

The NHIS-HEALS database included all medical expenditures for participants covered under the NHI from baseline to end of observation or death. The medical expenditures included copayments by beneficiaries and reimbursement costs by the insurer for all medical treatments including outpatient, inpatient, or emergency department visits under the NHI.

The incremental medical expenditures or CCI score ratios in the obesity I, II, and III groups were calculated as follows. The 11-year changes in medical expenditures or CCI scores were calculated for each obesity level. And the differences in changes between each obesity level and those of the normal weight group were divided by the 11-year change in the normal weight group and presented as percentages.

#### Ten-year disease incidence (2004–2013)

The incidences of individual diseases associated with obesity, such as congestive heart failure (CHF), cerebrovascular disease, chronic liver disease, uncomplicated or complicated diabetes mellitus (DM), hypertension, and depression were observed. For participants without an individual disease in 2002–2003, the development of an individual disease was confirmed based on the presence of diagnosis codes in 2004–2013.

### Data analysis

Continuous data are presented as means and standard deviation (SD), while categorical data are shown as frequencies. Mean and SD of CCI scores and medical expenditures at baseline (2002–2003) and at the end of the observation period (2012–2013) are presented according to BMI categories at baseline in entire population set. Data were also included from participants who remained in the same BMI categories at baseline and at the end of the observation period (consistent BMI level population set). Individuals with medical records in 2002 underwent follow-up until 2012, while those with data in 2003 were followed until 2013. Thus, 11 years of data were collected. To observe the association between disease incidence and obesity level, adjusted hazard ratio (aHR) after adjustments for age, sex, income level, and CCI score were also calculated using time-to-event hazard model. Death event or the end of study period was considered as censoring in the disease incidence analysis.

To investigate the impact of BMI on medical expenditures after adjusting for confounding variables such as demographic information and comorbidities, a generalized linear model (GLM) was used. Log link and gamma distributions were selected in the GLM to reflect the right-skewed distribution of the 11 years of medical expenditures [22]. We considered follow-up time as on offset variable because participants had different follow-up days owing to death. The dependent variable for medical expenditure in GLM was the ratio of 2012/3 to 2002/3. Cost ratio and 95% confidence intervals (CIs) using the GLM model are presented.

Data were analysed using SAS 9.4 (SAS Institute Inc., Seoul, Korea).

## Results

The entire population set included 496,469 individuals with BMI data in 2002–2003. A total of 214,477 people who remained in the same BMI category from baseline (2002–2003) to the end of the observation period (2012–2013) were selected as the consistent BMI level population set. The percentages of participants according to BMI category were 2.3% (underweight), 35.3% (normal weight), 27.3% (overweight), 32.3% (obesity I), 2.7% (obesity II), and 0.2% (obesity III).

Entire participants’ demographic, socioeconomic, and clinical data according to BMI category are shown in Table 1. The average age was slightly lower in higher BMI categories. Whereas the average age of the underweight group (<18.5 kg/m^2^) was 56.3 years (SD 11.8), it was 52.6 years (SD 9.1) in the very severe obesity group (35–59.99 kg/m^2^). The baseline CCI score was higher in the underweight (BMI <18.5 kg/m^2^) and overweight and obesity (≥23 kg/m^2^) groups compared to the score in the normal weight (18.5–22.99 kg/m^2^) group. The CCI score increased with increased BMI in the overweight and obese group. The prevalence of hypertension and depression according to BMI category showed similar trends to those of CCI scores. In addition, the descriptive statistics for the BMI level consistent population set were presented in S2 Table.

**Table 1:** Baseline characteristics of entire population.

Variables, N (%)	Total	Underweight* (<18.5 kg/m^2^)	Normal weight* (18.5–22.99 kg/m^2^)	Overweight* (23–24.99 kg/m^2^)	Obesity I* (25–29.99 kg/m^2^)	Obesity II* (30–34.99 kg/m^2^)	Obesity III* (35–59.99 kg/m^2^)
Total	496,469	11,233 (2.3%)	175,253 (35.3%)	135,341 (27.3%)	160,319 (32.3%)	13,453 (2.7%)	870 (0.2%)
Year							
2002	284,700	6,136 (2.2%)	100,906 (35.4%)	78,227 (27.5%)	91,775 (32.2%)	7,213 (2.5%)	443 (0.2%)
2003	211,769	5,097 (2.4%)	74,347 (35.1%)	57,114 (27.0%)	68,544 (32.4%)	6,240 (2.9%)	427 (0.2%)
Sex							
Male	267,824	6,106 (2.3%)	90,421 (33.8%)	75,835 (28.3%)	89,722 (33.5%)	5,490 (2.0%)	250 (0.1%)
Female	228,645	5,127 (2.2%)	84,832 (37.1%)	59,506 (26.0%)	70,597 (30.9%)	7,963 (3.5%)	620 (0.3%)
Age (years), mean (SD)	52.5 (9.6)	56.3 (11.8)	52.3 (10.0)	52.3 (9.3)	52.6 (9.1)	53.0 (9.2)	52.6 (9.1)
40-<50	231,657	4,249 (1.8%)	86,789 (37.5%)	63,337 (27.3%)	71,163 (30.7%)	5,723 (2.5%)	396 (0.2%)
50-<60	139,979	2,226 (1.6%)	43,836 (31.3%)	39,759 (28.4%)	49,687 (35.5%)	4,218 (3.0%)	253 (0.2%)
60-<70	95,475	2,853 (3.0%)	32,359 (33.9%)	25,356 (26.6%)	31,851 (33.4%)	2,872 (3.0%)	184 (0.2%)
≥70	29,358	1,905 (6.5%)	12,269 (41.8%)	6,889 (23.5%)	7,618 (25.9%)	640 (2.2%)	37 (0.1%)
Income levels							
NHI district subscriber 1–2	24,516	1,021 (4.2%)	9,134 (37.3%)	5,905 (24.1%)	7,518 (30.7%)	882 (3.6%)	56 (0.2%)
NHI district subscriber 3–7	87,170	2,467 (2.8%)	31,325 (35.9%)	22,328 (25.6%)	27,974 (32.1%)	2,856 (3.3%)	220 (0.3%)
NHI district subscriber 8–10	78,670	1,283 (1.6%)	25,174 (32.0%)	22,071 (28.1%)	27,630 (35.1%)	2,377 (3.0%)	135 (0.2%)
NHI employee subscriber 1–2	54,299	1,196 (2.2%)	19,668 (36.2%)	14,631 (26.9%)	17,142 (31.6%)	1,563 (2.9%)	99 (0.2%)
NHI employee subscriber 3–7	110,055	2,384 (2.2%)	40,099 (36.4%)	29,854 (27.1%)	34,666 (31.5%)	2,865 (2.6%)	187 (0.2%)
NHI employee subscriber 8–10	141,271	2,855 (2.0%)	49,681 (35.2%)	40,438 (28.6%)	45,229 (32.0%)	2,899 (2.1%)	169 (0.1%)
Medical aid	488	27 (5.5%)	172 (35.2%)	114 (23.4%)	160 (32.8%)	11 (2.3%)	4 (0.8%)
CCI score, mean (SD)	0.98 (1.27)	1.02 (1.26)	0.90 (1.20)	0.97 (1.26)	1.06 (1.33)	1.26 (1.48)	1.50 (1.61)
Other diseases not included in CCI							
Hypertension	112,345	1,676 (1.5%)	27,542 (24.5%)	29,876 (26.6%)	47,233 (42.0%)	5,567 (5.0%)	451 (0.4%)

* Western criteria are presented in parentheses by the near Asian criteria: Underweight (Underweight), Normal weight (Normal weight), Overweight (Normal weight), Obesity I (Overweight), Obesity II (Obesity I), and Obesity III (Obesity II).

CCI: Charlson Comorbidity Index, NHI: National Health Insurance, SD: standard deviation

**Fig 1:**
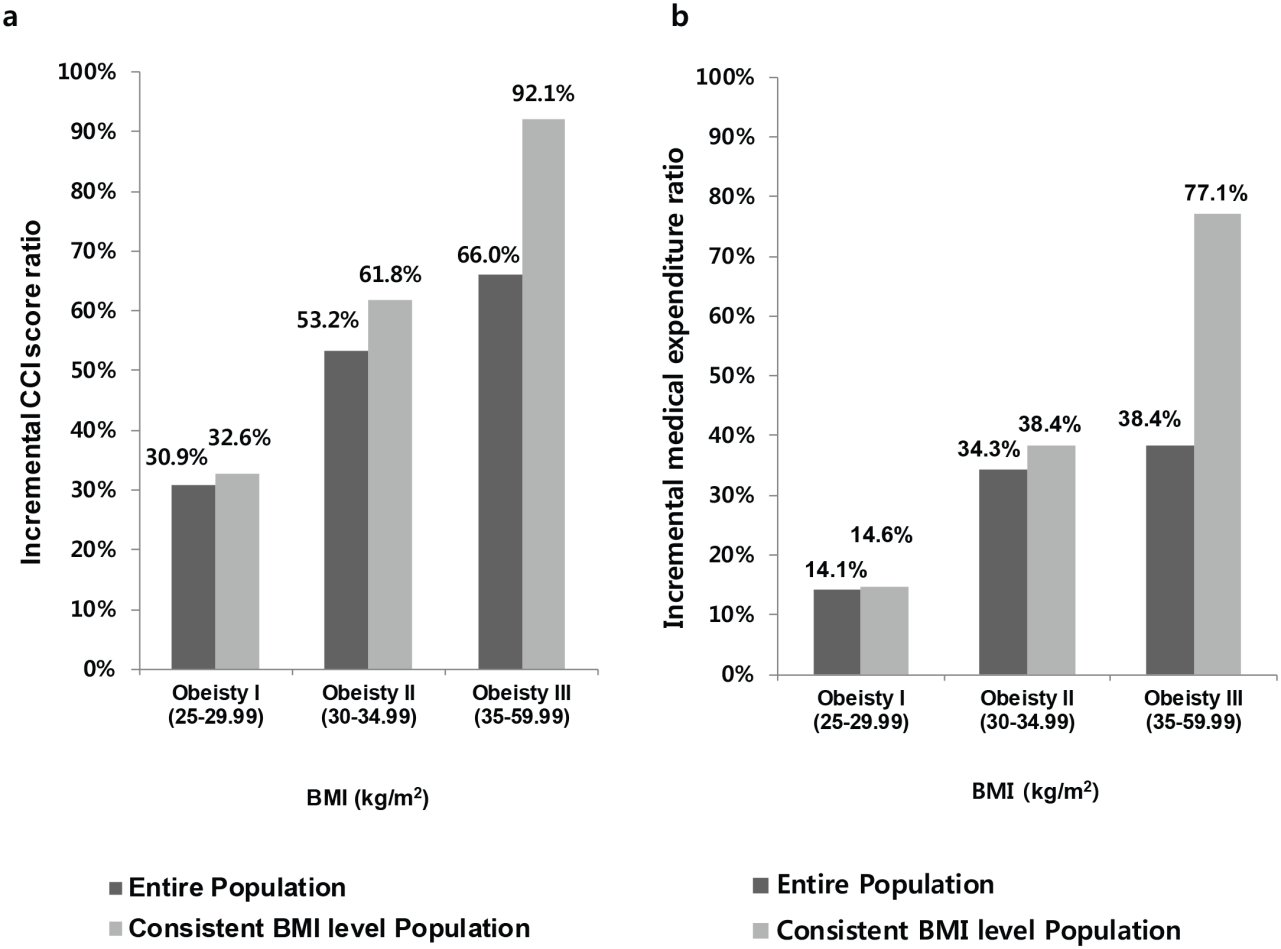
Incremental Charlson Comorbidity Index (CCI) score ratios and medical expenditures ratio over 11 years. **Comparison of 2002–2003 and 2012–2013 for entire population set (participants with available BMI data in 2002–2003) and consistent BMI level population set (participants who remained in their baseline BMI categories in 2012–2013) according to BMI a. Incremental CCI score ratio based on normal weight b. Incremental medical expenditure ratio based on normal weight**. * Incremental CCI or medical expenditure ratio = (11-year change of each obesity level– 11-year change in normal weight) / (11-year change in normal weight) *100.

The change in average CCI score over 11 years was 0.94 for the normal weight group (0.90 in 2002–2003 and 1.84 in 2012–2013) in the entire population (participants with BMI data in 2002–2003), while those in the overweight and obesity groups were 1.07 to 1.56. This trend was similar for the consistent BMI level population set (participants who remained in the same BMI category as in 2012–2013) (Table 2). The incremental CCI score ratios in the obesity I to III groups were 30.9% to 66.0% in the entire population set and 32.6% to 92.1% in the consistent BMI level population set compared to the normal weight group (Fig 1a).

**Table 2:** Change in Charlson Comorbidity Index (CCI) scores and medical expenditures between 2002–2003 and 2012–2013.

BMI^#^	Entire population set*				Consistent BMI level population set†			
	N	2002–2003 Mean (SD)	2012–2013 Mean (SD)	Change of mean score	N	2002–2003 Mean (SD)	2012–2013 Mean (SD)	Change of mean score
Charlson Comorbidity Index	496,469	0.98 (1.27)	2.07 (2.10)	1.09	214,477	0.91 (1.17)	1.93 (1.92)	1.02
Underweight (<18.5 kg/m^2^)	11,233	1.02 (1.26)	1.84 (2.05)	0.82	3,088	0.87 (1.09)	1.77 (1.88)	0.90
Normal weight (18.5–22.99 kg/m^2^)	175,253	0.90 (1.20)	1.84 (2.00)	0.94	85,725	0.84 (1.11)	1.73 (1.84)	0.89
Overweight (23–24.99 kg/m^2^)	135,341	0.97 (1.26)	2.04 (2.08)	1.07	45,768	0.89 (1.15)	1.88 (1.86)	0.99
Obesity I (25–29.99 kg/m^2^)	160,319	1.06 (1.33)	2.29 (2.18)	1.23	75,286	0.98 (1.24)	2.16 (1.99)	1.18
Obesity II (30–34.99 kg/m^2^)	13,453	1.26 (1.48)	2.70 (2.24)	1.44	4,395	1.14 (1.35)	2.58 (2.06)	1.44
Obesity III (35–59.99 kg/m^2^)	870	1.50 (1.61)	3.06 (2.56)	1.56	215	1.38 (1.52)	3.09 (2.32)	1.71
Medical cost (1,000 won)‡	496,469	541 (1,173)	2,541 (6,284)	2,000	214,477	475 (903)	1,916 (3,915)	1,441
Underweight (<18.5 kg/m^2^)	11,233	611 (1,580)	2,916 (7,373)	2,305	3,088	458 (1,016)	2,175 (4,475)	1,717
Normal weight (18.5–22.99 kg/m^2^)	175,253	511 (1,195)	2,375 (6,067)	1,864	85,725	444 (842)	1,802 (3,916)	1,358
Overweight (23–24.99 kg/m^2^)	135,341	528 (1,118)	2,477 (6,307)	1,949	45,768	458 (880)	1,798 (3,674)	1,340
Obesity I (25–29.99 kg/m^2^)	160,319	569 (1,151)	2,695 (6,354)	2,126	75,286	513 (964)	2,069 (3,991)	1,556
Obesity II (30–34.99 kg/m^2^)	13,453	664 (1,219)	3,168 (6,955)	2,504	4,395	598 (1,017)	2,477 (4,275)	1,879
Obesity III (35–59.99 kg/m^2^)	870	789 (1,592)	3,368 (6,293)	2,579	215	806 (1,712)	3,211 (6,425)	2,405

*Entire population: participants with available BMI data in 2002–2003

^†^Population with consistent BMI level for 11 years: participants who remained in their baseline BMI category in 2012–2013

^‡^1,000 South Korea Won = 0.92 US$ (based on 30 November 2017)

^**#**^Western criteria are presented in parentheses by the near Asian criteria: Underweight (Underweight), Normal weight (Normal weight), Overweight (Normal weight), Obesity I (Overweight), Obesity II (Obesity I), and Obesity III (Obesity II).

BMI: body mass index, SD: standard deviation

The change in average medical expenditures over 11 years was larger in the underweight and obesity groups (Table 2) and was greater with increasing obesity level. This trend was also similar between the entire and consistent BMI level population sets. The incremental medical expenditure ratios for the obesity I, II, and III groups were 14.1%, 34.3%, and 38.4%, respectively, in the entire population set and 14.6%, 38.4%, and 77.1% in the consistent BMI level population set (Fig 1b). The medical expenditures in the follow-up period (maximum 11 years) according to BMI category were calculated after adjusting for sex, age, income level, and comorbidities (CCI score, hypertension, and depression) using the GLM model with log link and gamma distribution (Table 3). The expenditures were significantly higher in the obese groups (obesity I, obesity II, and obesity III) compared to those of the normal weight group in the entire or consistent BMI level population sets. Individuals with BMI ≥35 kg/m^2^ spent about 1.40 times more than did the reference group of normal weight individuals in the consistent BMI level population set.

**Table 3:** Eleven-year medical expenditure ratios by BMI category after adjustments.

Variables (Baseline value in 2002–2003)	Entire population set* (n = 496,469)		Consistent BMI level population set† (n = 214,477)	
	11-year medical expenditure ratio	95% confidence interval	11-year medical expenditure ratio	95% confidence interval
Baseline BMI^#^				
Underweight (<18.5 kg/m^2^)	1.10	1.08–1.12	1.04	1.01–1.07
Normal weight (18.5–22.99 kg/m^2^)	1		1	
Overweight (23–24.99 kg/m^2^)	1.00	0.99–1.01	1.02	1.01–1.03
Obesity I (25–29.99 kg/m^2^)	1.04	1.03–1.05	1.10	1.09–1.11
Obesity II (30–34.99 kg/m^2^)	1.13	1.11–1.15	1.21	1.18–1.24
Obesity III (35–59.99 kg/m^2^)	1.20	1.12–1.28	1.40	1.25–1.57
Sex				
Male	1		1	
Female	1.00	0.99–1.01	1.17	1.16–1.18
Age (years)				
40-<50	1		1	
50-<60	1.49	1.48–1.50	1.33	1.32–1.35
60-<70	2.20	2.19–2.22	1.84	1.82–1.86
≥70	2.73	2.70–2.77	2.13	2.08–2.18
Income levels				
NHI district subscriber 1–2	1		1	
NHI district subscriber 3–7	0.97	0.95–0.98	1.00	0.98–1.02
NHI district subscriber 8–10	0.90	0.88–0.91	0.93	0.91–0.95
NHI employee subscriber 1–2	0.87	0.86–0.89	0.85	0.84–0.87
NHI employee subscriber 3–7	0.86	0.84–0.87	0.88	0.87–0.90
NHI employee subscriber 8–10	0.79	0.78–0.80	0.83	0.81–0.85
Medical aid	1.40	1.28–1.53	1.44	1.22–1.70
CCI score				
0	1		1	
1	1.26	1.25–1.27	1.31	1.30–1.32
2	1.52	1.50–1.53	1.60	1.58–1.61
3	1.78	1.75–1.80	1.83	1.80–1.86
≥4	2.39	2.35–2.42	2.20	2.16–2.25
Other diseases not included in CCI				
Hypertension				
No	1		1	
Yes	1.29	1.28–1.30	1.27	1.25–1.28
Depression				
No	1		1	
Yes	1.28	1.26–1.30	1.32	1.29–1.34

* Entire population: participants with available BMI data in 2002–2003

^†^ Population with consistent BMI level for 11 years: participants who remained in their baseline BMI categories in 2012–2013

^**#**^ Western criteria are presented in parentheses by the near Asian criteria: Underweight (Underweight), Normal weight (Normal weight), Overweight (Normal weight), Obesity I (Overweight), Obesity II (Obesity I), and Obesity III (Obesity II).

BMI: body mass index, CCI: Charlson Comorbidity index, NHI: National Health Insurance

Changes in CCI scores and medical expenditures in the subgroup analysis of men or women revealed that individuals with higher obesity levels showed worse health outcome in both groups (S3 Table). Medical expenditures after adjustments for confounding variables were significantly higher in the overweight and obese groups (obesity I, obesity II, and obesity III) in women, while they were significantly higher in the obesity II and obesity III groups in men (S4 Table).

**Fig 2:**
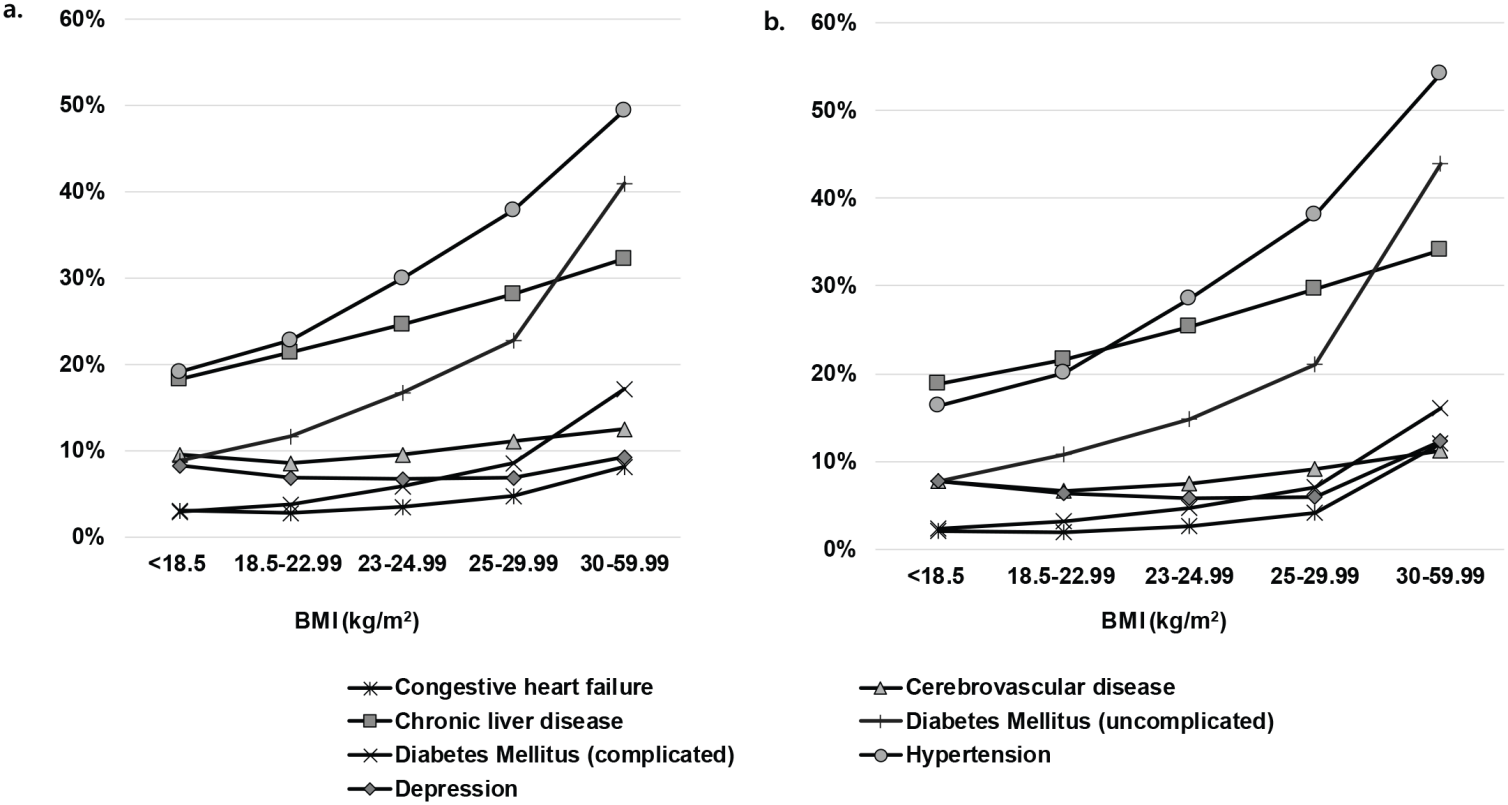
Disease incidence over 10 years (2004–2013) a. Entire population set (participants with available BMI data in 2002–2003) b. Consistent BMI level population set (participants who remained in their baseline BMI categories in 2012–2013).

Among participants without individual disease in 2002–2003, the incidence of several diseases increased in 2004–2013 with increasing BMI (Fig 2). Among participants with BMI ≥30 kg/m^2^ in the entire population set, the incidence rates of CHF, cerebrovascular disease, chronic liver disease, uncomplicated DM, complicated DM, hypertension, and depression were 8.1%, 12.5%, 32.2%, 41.0%, 17.2%, 49.4%, and 9.2%, respectively, over approximately 10 years. In the consistent BMI level population set, the incidence of CHF, chronic liver disease, uncomplicated DM, hypertension, and depression among participants with BMI ≥30 kg/m^2^ was higher than that in the entire population set. In particular, the highest aHRs of individuals with BMI ≥30 kg/m^2^ in the entire population in comparison to normal weight individuals were 2.33 (95% CI 2.26–2.39) for hypertension, 2.22 (95% CI 2.14–2.31) for complicated DM, 1.85 (95% CI 1.81–1.90) for uncomplicated DM, 1.58 (95% CI 1.51–1.65) for CHF, 1.30 (95% CI 1.27–1.34) for chronic liver disease, 1.08 (95% CI 1.05–1.12) for cerebrovascular disease, and 0.96 (95% CI 0.93–1.00) for depression. In the consistent BMI level population set, the aHRs for those diseases in individuals with BMI ≥30 kg/m^2^ were greater than those for in the entire population set. The aHR for depression was 1.18 (95% CI 1.00–1.39).

## Discussion

This study quantified the impact of BMI on medical expenditures over 11 years in a Korean population 40–79 years of age. The incremental medical expenditure ratio showed a dose-response relationship with increasing obesity level. The incremental medical expenditure ratios of the obesity I, II, and III groups over 11 years ranged from 14.6% to 77.1% compared to the normal weight group in subjects who were remained in the same category (i.e., the consistent BMI level population set). In the entire population set (i.e., those with BMI data in 2002–2003), similar results (14.1% to 38.4%) were observed even though the differences were slightly lower.

Furthermore, the impact of obesity on medical expenditures was quantified based on the follow-up periods using a GLM model. Because some individuals died during the observation period, this factor was also considered in analysis of medical expenditures. Obese individuals with BMI ≥30 kg/m^2^ had medical expenditures of 1.21–1.40 times those of normal weight individuals after adjusting for age, sex, income level, and comorbidities in the consistent BMI level population set over approximately 11 years.

Our study results were in accordance with those of previous studies that were limited in estimating the attributable costs of obesity by the cross-sectional design [10–12]. In a previous systematic review, the respective incremental costs of overweight and obesity were 9.9% and 42.7% higher than those of normal weight [10]. It is very well known that obesity is a major cause of chronic diseases. Our study also showed that higher CCI scores with higher baseline BMI were associated with a greater change in CCI score after 11 years. Increases in CCI scores in the normal BMI group over 11 years may be explained by the effects of age. The incremental CCI score changes in the obese group over those of the normal group might be attributed to the effects of obesity on CCI. In the obesity III (35–59.99 kg/m^2^) group, the CCI score change was about twice that of the normal group. The consistent BMI level population set experienced a greater change in CCI score. In addition, our study determined the obesity-related disease incidence according to the increase in BMI level over 10 years. The aHRs for obesity-related disease in the overweight and obesity groups were significantly higher than that in the normal weight group. Even though these findings were in accordance with those of previous studies [6, 23–24], the results of our study are meaningful in that they show the long-term effects of prevalence or incidence of obesity-related comorbidities in an Asian population.

In the present study, no increased medical expenditure in the overweight group (23–24.99 kg/m^2^) was observed compared to the normal weight group (cost ratio 1.00, 95% CI 0.99–1.01 and 1.02, 95% CI 1.01–1.03 in the entire and consistent BMI level population sets, respectively) in both sexes. In a separate analysis of men and women, women showed increased medical expenditures in the overweight group, but this trend was not observed in men. Even though there was no difference in medical costs in the overweight group, the change in CCI score and disease incidence in this group also showed increasing trends when compared to those of the normal weight group. The findings in the overweight group were consistent with those of previous studies on mortality according to obesity level [25]. The overweight group may not show the adverse effects of final outcomes such as mortality or medical expenditure compared to the normal weight group, especially for men. Different BMI criteria for definition of obesity by sex should be discussed and studied in the future.

The results of the present study indicate that obesity influenced higher medical expenditures after adjustment for demographic factors and baseline chronic diseases. This finding may be explained by the incidence of additional diseases due to obesity, as observed in our study (Fig 2). This trend was more prominent in the consistent BMI level population set, which remained in the same BMI category. The medical expenditure or CCI score change showed similar trends in the entire and consistent BMI level population sets, even though the incremental medical expenditure and CCI score ratio in the BMI ≥30 kg/m^2^ group were slightly higher in the consistent BMI level population set. We could not fully examine weight gain or loss during the follow-up periods owing to data limitations. Indeed, when we compare the results between subgroups of consistent BMI and changed BMI (increase or decrease), the subgroup of BMI decrease showed higher CCI score and medical cost than that of consistent BMI, especially in normal BMI range (S5 and S6 Tables). Unintentional weight loss is regarded as a sign of serious illness [26]. In many epidemiological studies, weight loss was associated with high mortality risk, which was explained by the preexisting disease [27–29]. Thus, it was possible that the BMI change, particularly BMI decrease, could confound the association between baseline BMI and medical cost. However, through comparison of the entire and consistent BMI level population sets, adverse effects of obesity on medical expenditure or disease incidence or prevalence were robustly confirmed. The influence of weight gain or loss may have been present in the entire population set, but could not be observed in the consistent BMI level population set. When considering the higher cost ratio of obesity in the consistent BMI level population set, the impact of obesity after excluding confounding factors due to weight change is more obvious.

The findings of the study imply the negative health influence of persistent obesity. Recently, the prevalence of BMI ≥30 kg/m^2^ has been increasing in Korea [30]. Our study results suggest a dramatic increase in disease burden and medical costs due to obesity in the near future. Thus, health policy regarding obesity, especially severe obesity, is required.

The present study accomplished several notable achievements. First, our results showed long-term follow-up results for obesity-related comorbidities and medical expenditures according to BMI over approximately 10 years in an Asian region. This study was original and unique in that no previous study has attempted to investigate medical expenditure in relation to obesity level as well as the incidence or prevalence of obesity-related disease in South Korea. Second, the quantification of the impact of obesity on medical expenditures offered valuable insights. We analysed the medical expenditures associated with obesity after adjusting for age, sex, income level, and comorbidities. Most previous studies on medical costs due to obesity did not control for those confounding factors. By determining the attributable costs related to obesity, the importance of obesity prevention and improved intervention strategies for obesity management may be highlighted from a public health perspective. Third, the incremental ratios of CCI scores were comprehensively analysed. It is expected that CCI score will increase with age. Therefore, the change in CCI score in the obesity group was compared with that of the normal weight group, i.e., the “incremental CCI score ratio”. With this measurement, we identified the additional disease burden attributable to obesity without the effect of aging.

This study had several limitations. First, the analysis included only data at baseline and after 11 years, so BMI trends between these time points were not reflected. However, we attempted to maintain the reliability of the results by analysing a consistent BMI level population set of individuals who remained at their baseline BMI category after 11 years. Second, the medical costs in the present study only included health insurance reimbursement costs and copayment under the NHI scheme. So, we did not consider other costs including out-of-pocket payments by the uninsured from the patient perspective, or indirect costs from the societal perspective. Therefore, the absolute costs could be underestimated. However, we can estimate the relative value of obesity compared to normal weight. In this regard, the results of our study may be meaningful.

Further research into the impact of weight gain or loss on the cost and disease incidence or prevalence would be helpful to investigate various benefits or adverse effects on weight-related public health issues. Additional studies considering the comprehensive costs including indirect costs from the societal perspective would be beneficial to understand the costs attributable to obesity.

## Conclusions

The present study estimated the incremental medical expenditure and chronic disease burden due to obesity over an 11-year follow-up period in an Asian region. The results could be a reference in Asian countries such as South Korea, which may have similar trends in increasing obesity prevalence (BMI ≥30 kg/m^2^), and may provide evidence for the development of effective and sustainable obesity management strategies.

## Supporting information

S1 Table The proportion by BMI level in our database and KNHANES. (DOCX)

S2 Table Baseline characteristics of consistent BMI level population. (DOCX)

S3 Table Change in Charlson Comorbidity Index (CCI) scores and medical expenditures between 2002–2003 and 2012–2013 in men and women. (DOCX)

S4 Table Eleven-year medical expenditure ratios by BMI category and sex after adjustments. (DOCX)

S5 Table Change in Charlson Comorbidity Index (CCI) scores and medical expenditures between 2002–2003 and 2012–2013 in people whose BMI increased or decreased. (DOCX)

S6 Table Eleven-year medical expenditure ratios by BMI category after adjustments in people whose BMI increased or decreased. (DOCX)

S1 Fig Flow chart of study population selection. (TIF)
