# Supplementary material for: The impact of obesity and overweight on medical expenditures and disease incidence in Korea from 2002 to 2013
Source: PLoS One. 2018 May 10;13(5):e0197057. doi: 10.1371/journal.pone.0197057 (PMC5944944; doi:10.1371/journal.pone.0197057)
Supplement: S1 Table — (DOCX) [file pone.0197057.s001.docx]

**S1 Table. The proportion by BMI level in our database and KNHANES**

|  | NHIS-HEALS  (2002-2003) | KNHANES II*  (2001) | | KNHANES III*  (2005) | |
| --- | --- | --- | --- | --- | --- |
|  | N (%) | N (%) | SE (95% CI)^†^ | N (%) | SE (95% CI)^†^ |
| BMI |  |  |  |  |  |
| <18.5 kg/㎡ | 11,233 (2.3%) | 99 (2.7%) | 0.3 (2.1-3.2) | 95 (2.6%) | 0.3 (2.0-3.1) |
| 18.5-22.99 kg/㎡ | 175,253 (35.3%) | 1,278 (35.1%) | 1.0 (33.1-37.2) | 1,131 (32.1%) | 0.9 (30.4-33.9) |
| 23-24.99 kg/㎡ | 135,341 (27.3%) | 985 (25.9%) | 0.9 (24.1-27.7) | 958 (27.7%) | 0.8 (26.1-29.3) |
| 25-29.99 kg/㎡ | 160,319 (32.3%) | 1,215 (32.7%) | 1.0 (30.9-34.6) | 1,185 (34.3%) | 1.0 (32.3-36.4) |
| 30-34.99 kg/㎡ | 13,453 (2.7%) | 117 (3.3%) | 0.3 (2.6-4.0) | 105 (3.2%) | 0.4 (2.5-4.0) |
| 35-59.99 kg/㎡ | 870 (0.2%) | 9 (0.3%) | 0.1 (0.1-0.5) | 5 (0.1%) | 0.1 (0.0-0.2) |
| Total | 496,469 (100.0%) | 3,703 (100.0%) | - | 3,479 (100.0%) | - |

* This results was obtained from subjects aged 40-79 among the participants of physical examination of KNHANES II (N=9,702) and KNHANES III (N=7,597). We presented unweighted sample size and weighted percent using KNHANES sample weight.

^†^ Standard error and 95% confidence interval.

BMI: body mass index
