# Supplementary material for: The impact of obesity and overweight on medical expenditures and disease incidence in Korea from 2002 to 2013
Source: PLoS One. 2018 May 10;13(5):e0197057. doi: 10.1371/journal.pone.0197057 (PMC5944944; doi:10.1371/journal.pone.0197057)
Supplement: S2 Table — (DOCX) [file pone.0197057.s002.docx]

S2 Table. Baseline characteristics of consistent BMI level population

| Variables, N (%) | Total | Underweight (<18.5 kg/m^2^) | Normal weight (18.5–22.99 kg/m^2^) | Overweight  (23–24.99 kg/m^2^) | Obesity I  (25–29.99 kg/m^2^) | Obesity II  (30–34.99 kg/m^2^) | Obesity III  (35-59.99 kg/m^2^) |
| --- | --- | --- | --- | --- | --- | --- | --- |
| Total | 214,477 | 3,088 (1.4%) | 85,725 (40.0%) | 45,768 (21.3%) | 75,286 (35.1%) | 4,395 (2.1%) | 215 (0.1%) |
| Year |  |  |  |  |  |  |  |
| 2002 | 129,842 | 1,848 (1.4%) | 51,687 (39.8%) | 28,073 (21.6%) | 45,604 (35.1%) | 2,507 (1.9%) | 123 (0.1%) |
| 2003 | 84,635 | 1,240 (1.5%) | 34,038 (40.2%) | 17,695 (20.9%) | 29,682 (35.1%) | 1,888 (2.2%) | 92 (0.1%) |
| Sex |  |  |  |  |  |  |  |
| Male | 116,210 | 1,619 (1.4%) | 42,288 (36.4%) | 26,629 (22.9%) | 43,762 (37.7%) | 1,848 (1.6%) | 64 (0.1%) |
| Female | 98,267 | 1,469 (1.5%) | 43,437 (44.2%) | 19,139 (19.5%) | 31,524 (32.1%) | 2,547 (2.6%) | 151 (0.2%) |
| Age (years), mean (SD) | 50.61 (8.49) | 53.5 (10.26) | 50.38 (8.81) | 50.42 (8.19) | 50.84 (8.19) | 51.03 (8.20) | 50.05 (7.83) |
| 40-<50 | 116,721 | 1,398 (1.2%) | 48,918 (41.9%) | 25,027 (21.4%) | 39,012 (33.4%) | 2,244 (1.9%) | 122 (0.1%) |
| 50-<60 | 56,549 | 662 (1.2%) | 20,051 (35.5%) | 12,599 (22.3%) | 21,884 (38.7%) | 1,292 (2.3%) | 61 (0.1%) |
| 60-<70 | 36,269 | 801 (2.2%) | 14,214 (39.2%) | 7,315 (20.2%) | 13,111 (36.2%) | 798 (2.2%) | 30 (0.1%) |
| ≥70 | 4,938 | 227 (4.6%) | 2,542 (51.5%) | 827 (16.8%) | 1,279 (25.9%) | 61 (1.2%) | 2 (0.0%) |
| Income levels |  |  |  |  |  |  |  |
| NHI district subscriber 1-2 | 7,530 | 174 (2.3%) | 3,164 (42.0%) | 1,353 (18.0%) | 2,603 (34.6%) | 220 (2.9%) | 16 (0.2%) |
| NHI district subscriber 3-7 | 34,660 | 594 (1.7%) | 14,141 (40.8%) | 6,790 (19.6%) | 12,152 (35.1%) | 924 (2.7%) | 59 (0.2%) |
| NHI district subscriber 8-10 | 34,380 | 386 (1.1%) | 12,598 (36.6%) | 7,471 (21.7%) | 13,132 (38.2%) | 762 (2.2%) | 31 (0.1%) |
| NHI employee subscriber 1-2 | 22,880 | 371 (1.6%) | 9,440 (41.3%) | 4,680 (20.5%) | 7,851 (34.3%) | 515 (2.3%) | 23 (0.1%) |
| NHI employee subscriber 3-7 | 48,847 | 688 (1.4%) | 20,163 (41.3%) | 10,281 (21.1%) | 16,707 (34.2%) | 966 (2.0%) | 42 (0.1%) |
| NHI employee subscriber 8-10 | 66,081 | 874 (1.3%) | 26,172 (39.6%) | 15,179 (23.0%) | 22,808 (34.5%) | 1,006 (1.5%) | 42 (0.1%) |
| Medical aid | 99 | 1 (1.0%) | 47 (47.5%) | 14 (14.1%) | 33 (33.3%) | 2 (2.0%) | 2 (2.0%) |
| CCI score, mean (SD) | 0.91 (1.17) | 0.87 (1.09) | 0.84 (1.11) | 0.89 (1.15) | 0.98 (1.24) | 1.14 (1.35) | 1.38 (1.52) |
| Other diseases not included in CCI |  |  |  |  |  |  |  |
| Hypertension | 40,953 | 281 (0.7%) | 10,753 (26.3%) | 8,363 (20.4%) | 19,744 (48.2%) | 1,695 (4.1%) | 117 (0.3%) |
| Depression | 7,939 | 125 (1.6%) | 3,231 (40.7%) | 1,577 (19.9%) | 2,791 (35.2%) | 204 (2.6%) | 11 (0.1%) |

* Western criteria are presented in parentheses by the near Asian criteria: Underweight (Underweight), Normal weight (Normal weight), Overweight (Normal weight), Obesity I (Overweight), Obesity II (Obesity I), and Obesity III (Obesity II).

CCI: Charlson Comorbidity Index, NHI: National Health Insurance, SD: standard deviation
