# Supplementary material for: The impact of obesity and overweight on medical expenditures and disease incidence in Korea from 2002 to 2013
Source: PLoS One. 2018 May 10;13(5):e0197057. doi: 10.1371/journal.pone.0197057 (PMC5944944; doi:10.1371/journal.pone.0197057)
Supplement: S3 Table — (DOCX) [file pone.0197057.s003.docx]

**S3 Table. Change in Charlson comorbidity index (CCI) scores and medical expenditures between 2002-2003 and 2012-2013 in men and women**

| BMI**^#^** | | Entire population set* | | | | | | | | Consistent BMI level population set† | | | | | |
| --- | --- | --- | --- | --- | --- | --- | --- | --- | --- | --- | --- | --- | --- | --- | --- |
|  |  | N | | 2002-2003 Mean (SD) | | 2012-2013 Mean (SD) | | Change of mean score | | N | | 2002-2003 Mean (SD) | | 2012-2013 Mean (SD) | Change of mean score |
| Charlson comorbidity index | |  | |  | |  | |  | |  | |  | |  |  |
| Men | | 267,824 | | 0.86 (1.20) | | 2.00 (2.17) | | 1.14 | | 116,210 | | 0.79 (1.10) | | 1.86 (1.96) | 1.07 |
| Underweight (<18.5 kg/m^2^) | | 6,106 | | 0.99 (1.27) | | 1.89 (2.19) | | 0.90 | | 1,619 | | 0.79 (1.08) | | 1.84 (2.04) | 1.05 |
| Normal weight (18.5-22.99 kg/m^2^) | | 90,421 | | 0.83 (1.17) | | 1.84 (2.14) | | 1.01 | | 42,288 | | 0.75 (1.05) | | 1.72 (1.95) | 0.97 |
| Overweight (23-24.99 kg/m^2^) | | 75,835 | | 0.85 (1.19) | | 1.97 (2.15) | | 1.12 | | 26,629 | | 0.79 (1.10) | | 1.80 (1.89) | 1.01 |
| Obesity I (25-29.99 kg/m^2^) | | 89,722 | | 0.89 (1.22) | | 2.14 (2.20) | | 1.25 | | 43,762 | | 0.83 (1.14) | | 2.01 (1.99) | 1.18 |
| Obesity II (30-34.99 kg/m^2^) | | 5,490 | | 0.95 (1.28) | | 2.51 (2.27) | | 1.56 | | 1,848 | | 0.88 (1.21) | | 2.42 (2.06) | 1.54 |
| Obesity III (35-59.99 kg/m^2^) | | 250 | | 1.12 (1.28) | | 3.02 (2.95) | | 1.90 | | 64 | | 1.13 (1.21) | | 3.33 (2.38) | 2.20 |
| Women | 228,645 | | 1.13 (1.34) | | 2.15 (2.02) | | 1.02 | | 98,267 | | 1.04 (1.24) | | 2.02 (1.86) | | 0.98 |
| Underweight (<18.5 kg/m^2^) | 5,127 | | 1.05 (1.24) | | 1.78 (1.89) | | 0.73 | | 1,469 | | 0.96 (1.10) | | 1.70 (1.69) | | 0.74 |
| Normal weight (18.5-22.99 kg/m^2^) | 84,832 | | 0.99 (1.23) | | 1.83 (1.84) | | 0.84 | | 43,437 | | 0.92 (1.16) | | 1.75 (1.73) | | 0.83 |
| Overweight (23-24.99 kg/m^2^) | 59,506 | | 1.12 (1.32) | | 2.13 (2.00) | | 1.01 | | 19,139 | | 1.02 (1.22) | | 2.00 (1.82) | | 0.98 |
| Obesity I (25-29.99 kg/m^2^) | 70,597 | | 1.27 (1.42) | | 2.47 (2.13) | | 1.20 | | 31,524 | | 1.19 (1.33) | | 2.36 (1.97) | | 1.17 |
| Obesity II (30-34.99 kg/m^2^) | 7,963 | | 1.47 (1.57) | | 2.83 (2.22) | | 1.36 | | 2,547 | | 1.32 (1.42) | | 2.71 (2.05) | | 1.39 |
| Obesity III (35-59.99 kg/m^2^) | 620 | | 1.66 (1.70) | | 3.07 (2.38) | | 1.41 | | 151 | | 1.49 (1.62) | | 2.99 (2.29) | | 1.50 |
| Medical cost (1,000 won)‡ | |  | |  | |  | |  | |  | |  | |  |  |
| Men | | 267,824 | | 460 (1,176) | | 2,391 (6,448) | | 1,931 | | 116,210 | | 402 (896) | | 1,781 (4,160) | 1,379 |
| Underweight (<18.5 kg/m^2^) | | 6,106 | | 586 (1,631) | | 3,033 (7,864) | | 2,447 | | 1,619 | | 415 (1,230) | | 2,191 (4,449) | 1,776 |
| Normal weight (18.5-22.99 kg/m^2^) | | 90,421 | | 461 (1,282) | | 2,422 (6,521) | | 1,961 | | 42,288 | | 388 (884) | | 1,820 (4,482) | 1,432 |
| Overweight (23-24.99 kg/m^2^) | | 75,835 | | 452 (1,133) | | 2,315 (6,338) | | 1,863 | | 26,629 | | 398 (911) | | 1,651 (3,746) | 1,253 |
| Obesity I (25-29.99 kg/m^2^) | | 89,722 | | 456 (1,073) | | 2,379 (6,357) | | 1,923 | | 43,762 | | 415 (885) | | 1,801 (4,077) | 1,386 |
| Obesity II (30-34.99 kg/m^2^) | | 5,490 | | 449 (924) | | 2,532 (6,718) | | 2,083 | | 1,848 | | 422 (789) | | 1,902 (3,735) | 1,480 |
| Obesity III (35-59.99 kg/m^2^) | | 250 | | 603 (1,146) | | 3,171 (7,315) | | 2,568 | | 64 | | 747 (1,723) | | 2,525 (4,741) | 1,778 |
| Women | 228,645 | | 637 (1,161) | | 2,712 (6,089) | | 2,075 | | 98,267 | | 562 (904) | | 2,075 (3,598) | | 1,513 |
| Underweight (<18.5 kg/m^2^) | 5,127 | | 642 (1,517) | | 2,795 (6,826) | | 2,153 | | 1,469 | | 506 (705) | | 2,157 (4,505) | | 1,651 |
| Normal weight (18.5-22.99 kg/m^2^) | 84,832 | | 564 (1,093) | | 2,326 (5,569) | | 1,762 | | 43,437 | | 498 (796) | | 1,785 (3,271) | | 1,287 |
| Overweight (23-24.99 kg/m^2^) | 59,506 | | 624 (1,092) | | 2,678 (6,263) | | 2,054 | | 19,139 | | 541 (827) | | 2,003 (3,562) | | 1,462 |
| Obesity I (25-29.99 kg/m^2^) | 70,597 | | 714 (1,227) | | 3,092 (6,328) | | 2,378 | | 31,524 | | 650 (1,049) | | 2,441 (3,837) | | 1,791 |
| Obesity II (30-34.99 kg/m^2^) | 7,963 | | 812 (1,366) | | 3,603 (7,080) | | 2,791 | | 2,547 | | 725 (1,138) | | 2,893 (4,584) | | 2,168 |
| Obesity III (35-59.99 kg/m^2^) | 620 | | 864 (1,735) | | 3,447 (5,835) | | 2,583 | | 151 | | 831 (1,712) | | 3,502 (7,011) | | 2,671 |

* Entire population: participants with available BMI data in 2002-2003

†Population with consistent BMI level for 11 years: participants who remained in their baseline BMI category in 2012-2013

‡1,000 South Korea Won = 0.92 US$ (based on November 2017)

^#^The Western criteria were presented in parenthesis in the near Asian criteria: Underweight (Underweight), Normal weight (Normal weight), Overweight (Normal weight), Obesity I (Overweight), Obesity II (Obesity I), and Obesity III (Obesity II).

BMI: body mass index
