# Supplementary material for: The impact of obesity and overweight on medical expenditures and disease incidence in Korea from 2002 to 2013
Source: PLoS One. 2018 May 10;13(5):e0197057. doi: 10.1371/journal.pone.0197057 (PMC5944944; doi:10.1371/journal.pone.0197057)
Supplement: S4 Table — (DOCX) [file pone.0197057.s004.docx]

**S4 Table. Eleven-year medical expenditure ratios by BMI category and sex after adjustments**

| Variables  (Baseline value in 2002-2003) | Men | |  | | Women |  |  |  |
| --- | --- | --- | --- | --- | --- | --- | --- | --- |
|  | Entire population set*  (n =267,824) | | Consistent BMI level population set†  (n = 116,210) | | Entire population set*  (n = 228,645) | | Consistent BMI level population set†  (n =98,267) | |
|  | 11-year medical expenditure ratio | 95% Confidence interval | 11-year medical expenditure ratio | 95% Confidence interval | 11-year medical expenditure ratio | 95% Confidence interval | 11-year medical expenditure ratio | 95% Confidence interval |
| Baseline BMI**^#^** |  |  |  |  |  |  |  |  |
| Underweight (<18.5 kg/m^2^) | 1.09 | 1.06-1.12 | 1.02 | 0.98-1.07 | 1.09 | 1.06-1.12 | 1.05 | 1.01-1.09 |
| Normal weight (18.5-22.99 kg/m^2^) | 1 |  | 1 |  | 1 |  | 1 |  |
| Overweight (23-24.99 kg/m^2^) | 0.96 | 0.95-0.97 | 0.98 | 0.96-0.99 | 1.07 | 1.06-1.08 | 1.08 | 1.06-1.09 |
| Obesity I (25-29.99 kg/m^2^) | 1.00 | 0.99-1.01 | 1.06 | 1.04-1.07 | 1.14 | 1.13-1.15 | 1.19 | 1.18-1.20 |
| Obesity II (30-34.99 kg/m^2^) | 1.04 | 1.01-1.08 | 1.09 | 1.04-1.14 | 1.26 | 1.24-1.29 | 1.36 | 1.32-1.40 |
| Obesity III (35-59.99 kg/m^2^) | 1.15 | 1.01-1.31 | 1.26 | 1.01-1.58 | 1.28 | 1.19-1.37 | 1.53 | 1.35-1.73 |
| Age (years) |  |  |  |  |  |  |  |  |
| 40-<50 | 1 |  | 1 |  | 1 |  | 1 |  |
| 50-<60 | 1.61 | 1.60-1.63 | 1.39 | 1.37-1.40 | 1.33 | 1.32-1.34 | 1.26 | 1.24-1.27 |
| 60-<70 | 2.41 | 2.38-2.44 | 1.96 | 1.93-1.99 | 1.93 | 1.91-1.95 | 1.68 | 1.66-1.71 |
| ≥70 | 3.01 | 2.95-3.07 | 2.40 | 2.31-2.49 | 2.40 | 2.37-2.44 | 1.84 | 1.78-1.90 |
| Income levels |  |  |  |  |  |  |  |  |
| NHI district subscriber 1-2 | 1 |  | 1 |  | 1 |  | 1 |  |
| NHI district subscriber 3-7 | 0.94 | 0.92-0.96 | 1.01 | 0.97-1.05 | 0.96 | 0.94-0.98 | 0.97 | 0.95-0.99 |
| NHI district subscriber 8-10 | 0.84 | 0.82-0.86 | 0.90 | 0.86-0.93 | 0.90 | 0.89-0.92 | 0.92 | 0.90-0.94 |
| NHI employee subscriber 1-2 | 0.87 | 0.84-0.89 | 0.85 | 0.82-0.88 | 0.84 | 0.82-0.85 | 0.83 | 0.81-0.85 |
| NHI employee subscriber 3-7 | 0.81 | 0.79-0.82 | 0.85 | 0.82-0.88 | 0.88 | 0.87-0.90 | 0.90 | 0.88-0.93 |
| NHI employee subscriber 8-10 | 0.73 | 0.72-0.75 | 0.81 | 0.78-0.84 | 0.85 | 0.84-0.87 | 0.86 | 0.84-0.88 |
| Medical aid | 1.52 | 1.29-1.80 | 1.69 | 1.20-2.39 | 1.31 | 1.19-1.44 | 1.31 | 1.09-1.56 |
| CCI score |  |  |  |  |  |  |  |  |
| 0 | 1 |  | 1 |  | 1 |  | 1 |  |
| 1 | 1.26 | 1.25-1.27 | 1.33 | 1.32-1.35 | 1.25 | 1.24-1.26 | 1.27 | 1.26-1.29 |
| 2 | 1.53 | 1.51-1.55 | 1.64 | 1.62-1.67 | 1.50 | 1.48-1.51 | 1.53 | 1.51-1.55 |
| 3 | 1.84 | 1.81-1.88 | 1.93 | 1.88-1.98 | 1.71 | 1.69-1.74 | 1.72 | 1.69-1.76 |
| ≥4 | 2.59 | 2.53-2.65 | 2.44 | 2.36-2.52 | 2.22 | 2.18-2.26 | 2.00 | 1.95-2.05 |
| Other diseases not included in CCI |  |  |  |  |  |  |  |  |
| Hypertension |  |  |  |  |  |  |  |  |
| No | 1 |  | 1 |  | 1 |  | 1 |  |
| Yes | 1.33 | 1.31-1.34 | 1.32 | 1.30-1.34 | 1.26 | 1.24-1.27 | 1.21 | 1.19-1.22 |
| Depression |  |  |  |  |  |  |  |  |
| No | 1 |  | 1 |  | 1 |  | 1 |  |
| Yes | 1.30 | 1.27-1.33 | 1.36 | 1.31-1.41 | 1.28 | 1.26-1.30 | 1.31 | 1.28-1.34 |

* Entire population: participants with available BMI data in 2002-2003

†Population with consistent BMI level for 11 years: participants who remained in their baseline BMI category in 2012-2013

‡1,000 South Korea Won = 0.92 US$ (based on November 2017)

^#^The Western criteria were presented in parenthesis in the near Asian criteria: Underweight (Underweight), Normal weight (Normal weight), Overweight (Normal weight), Obesity I (Overweight), Obesity II (Obesity I), and Obesity III (Obesity II).

BMI: body mass index, CCI: Charlson comorbidity index, NHI: National Health Insurance
