# Supplementary material for: The impact of obesity and overweight on medical expenditures and disease incidence in Korea from 2002 to 2013
Source: PLoS One. 2018 May 10;13(5):e0197057. doi: 10.1371/journal.pone.0197057 (PMC5944944; doi:10.1371/journal.pone.0197057)
Supplement: S5 Table — (DOCX) [file pone.0197057.s005.docx]

**S5 Table.** **Change in Charlson Comorbidity Index (CCI) scores and medical expenditures between 2002-2003 and 2012-2013 in people whose BMI increased or decreased**

| Baseline BMI**^#^** | BMI increased people (n=58,936) | | | | BMI decreased people (n=62,066) | | | |
| --- | --- | --- | --- | --- | --- | --- | --- | --- |
|  | N | 2002-2003 Mean (SD) | 2012-2013 Mean (SD) | Change of mean score | N | 2002-2003 Mean (SD) | 2012-2013 Mean (SD) | Change of mean score |
| Charlson Comorbidity Index | 58,936 | 0.87 (1.14) | 1.87 (1.88) | 1.00 | 62,066 | 1.03 (1.29) | 2.29 (2.14) | 1.26 |
| Underweight (<18.5 kg/m^2^) | 2,986 | 0.87 (1.12) | 1.65 (1.86) | 0.78 |  |  |  |  |
| Normal weight (18.5–22.99 kg/m^2^) | 28,167 | 0.83 (1.1) | 1.77 (1.82) | 0.94 | 4,227 | 0.93 (1.17) | 2.14 (2.11) | 1.21 |
| Overweight (23–24.99 kg/m^2^) | 22,913 | 0.88 (1.15) | 1.91 (1.89) | 1.03 | 24,949 | 1 (1.26) | 2.16 (2.08) | 1.16 |
| Obesity I (25–29.99 kg/m^2^) | 4,546 | 1.05 (1.29) | 2.34 (2.05) | 1.29 | 28,755 | 1.05 (1.31) | 2.36 (2.17) | 1.31 |
| Obesity II (30–34.99 kg/m^2^) | 324 | 1.29 (1.47) | 2.77 (2.19) | 1.48 | 3,830 | 1.24 (1.46) | 2.73 (2.2) | 1.49 |
| Obesity III (35-59.99 kg/m^2^) |  |  |  |  | 305 | 1.43 (1.47) | 2.89 (2.24) | 1.46 |
| Medical cost (1,000 won)‡ | 58,936 | 485 (979) | 1,907 (3,915) | 1,422 | 62,066 | 539 (977) | 2,437 (5,096) | 1,898 |
| Underweight (<18.5 kg/m^2^) | 2,986 | 479 (1,123) | 1,991 (5,426) | 1,512 |  |  |  |  |
| Normal weight (18.5–22.99 kg/m^2^) | 28,167 | 470 (931) | 1,838 (3,798) | 1,368 | 4,227 | 507 (911) | 2,600 (5,063) | 2,093 |
| Overweight (23–24.99 kg/m^2^) | 22,913 | 475 (948) | 1,897 (3,782) | 1,422 | 24,949 | 530 (969) | 2,323 (5,048) | 1,793 |
| Obesity I (25–29.99 kg/m^2^) | 4,546 | 618 (1,251) | 2,266 (3,949) | 1,648 | 28,755 | 540 (974) | 2,466 (5,135) | 1,926 |
| Obesity II (30–34.99 kg/m^2^) | 324 | 714 (1,227) | 2,832 (5,366) | 2,118 | 3,830 | 608 (1,121) | 2,771 (5,232) | 2,163 |
| Obesity III (35-59.99 kg/m^2^) |  |  |  |  | 305 | 639 (851) | 2,577 (3,411) | 1,938 |

‡1,000 South Korea Won = 0.92 US$ (based on 30 November 2017)

**^#^**Western criteria are presented in parentheses by the near Asian criteria: Underweight (Underweight), Normal weight (Normal weight), Overweight (Normal weight), Obesity I (Overweight), Obesity II (Obesity I), and Obesity III (Obesity II).

BMI: body mass index, SD: standard deviation
