# Supplementary material for: The impact of obesity and overweight on medical expenditures and disease incidence in Korea from 2002 to 2013
Source: PLoS One. 2018 May 10;13(5):e0197057. doi: 10.1371/journal.pone.0197057 (PMC5944944; doi:10.1371/journal.pone.0197057)
Supplement: S6 Table — (DOCX) [file pone.0197057.s006.docx]

S6 Table. Eleven-year medical expenditure ratios by BMI category after adjustments in people whose BMI increased or decreased

| Variables  (Baseline value in 2002-2003) | BMI increased people (n=58,936) | | BMI decreased people (n=62,066) | |
| --- | --- | --- | --- | --- |
|  | 11-year medical expenditure ratio | 95% confidence interval | 11-year medical expenditure ratio | 95% confidence interval |
| Baseline BMI**^#^** |  |  |  |  |
| Underweight (<18.5 kg/m^2^) | 0.97 | 0.93-1.00 |  |  |
| Normal weight (18.5–22.99 kg/m^2^) | 1 |  | 1 |  |
| Overweight (23–24.99 kg/m^2^) | 1.02 | 1.01-1.04 | 0.97 | 0.95-1.00 |
| Obesity I (25–29.99 kg/m^2^) | 1.13 | 1.10-1.16 | 1.01 | 0.98-1.04 |
| Obesity II (30–34.99 kg/m^2^) | 1.34 | 1.22-1.47 | 1.09 | 1.05-1.13 |
| Obesity III (35-59.99 kg/m^2^) | - | - | 1.1 | 0.99-1.22 |
| Sex |  |  |  |  |
| Male | 1 |  | 1 |  |
| Female | 1.16 | 1.15-1.18 | 1.13 | 1.11-1.14 |
| Age (years) |  |  |  |  |
| 40-<50 | 1 |  | 1 |  |
| 50-<60 | 1.37 | 1.35-1.39 | 1.32 | 1.30-1.34 |
| 60-<70 | 1.84 | 1.81-1.88 | 1.76 | 1.73-1.79 |
| ≥70 | 1.99 | 1.89-2.10 | 1.99 | 1.92-2.07 |
| Income levels |  |  |  |  |
| NHI district subscriber 1-2 | 1 |  | 1 |  |
| NHI district subscriber 3-7 | 0.92 | 0.88-0.96 | 0.98 | 0.95-1.02 |
| NHI district subscriber 8-10 | 0.84 | 0.80-0.87 | 0.92 | 0.89-0.96 |
| NHI employee subscriber 1-2 | 0.77 | 0.74-0.80 | 0.88 | 0.85-0.91 |
| NHI employee subscriber 3-7 | 0.79 | 0.76-0.82 | 0.90 | 0.87-0.93 |
| NHI employee subscriber 8-10 | 0.71 | 0.69-0.74 | 0.87 | 0.84-0.90 |
| Medical aid | 1.24 | 0.94-1.62 | 1.48 | 1.14-1.92 |
| CCI score |  |  |  |  |
| 0 | 1 |  | 1 |  |
| 1 | 1.30 | 1.28-1.32 | 1.27 | 1.25-1.29 |
| 2 | 1.58 | 1.55-1.62 | 1.54 | 1.51-1.57 |
| 3 | 1.84 | 1.78-1.90 | 1.78 | 1.73-1.84 |
| ≥4 | 2.22 | 2.13-2.31 | 2.24 | 2.17-2.31 |
| Other diseases not included in CCI |  |  |  |  |
| Hypertension |  |  |  |  |
| No | 1 |  | 1 |  |
| Yes | 1.29 | 1.26-1.31 | 1.28 | 1.26-1.30 |
| Depression |  |  |  |  |
| No | 1 |  | 1 |  |
| Yes | 1.43 | 1.38-1.48 | 1.30 | 1.25-1.34 |

**^#^** Western criteria are presented in parentheses by the near Asian criteria: Underweight (Underweight), Normal weight (Normal weight), Overweight (Normal weight), Obesity I (Overweight), Obesity II (Obesity I), and Obesity III (Obesity II).

BMI: body mass index, CCI: Charlson Comorbidity index, NHI: National Health Insurance
