# Supplementary figures and images for: The impact of obesity and overweight on medical expenditures and disease incidence in Korea from 2002 to 2013
Source: PLoS One. 2018 May 10;13(5):e0197057. doi: 10.1371/journal.pone.0197057 (PMC5944944; doi:10.1371/journal.pone.0197057)

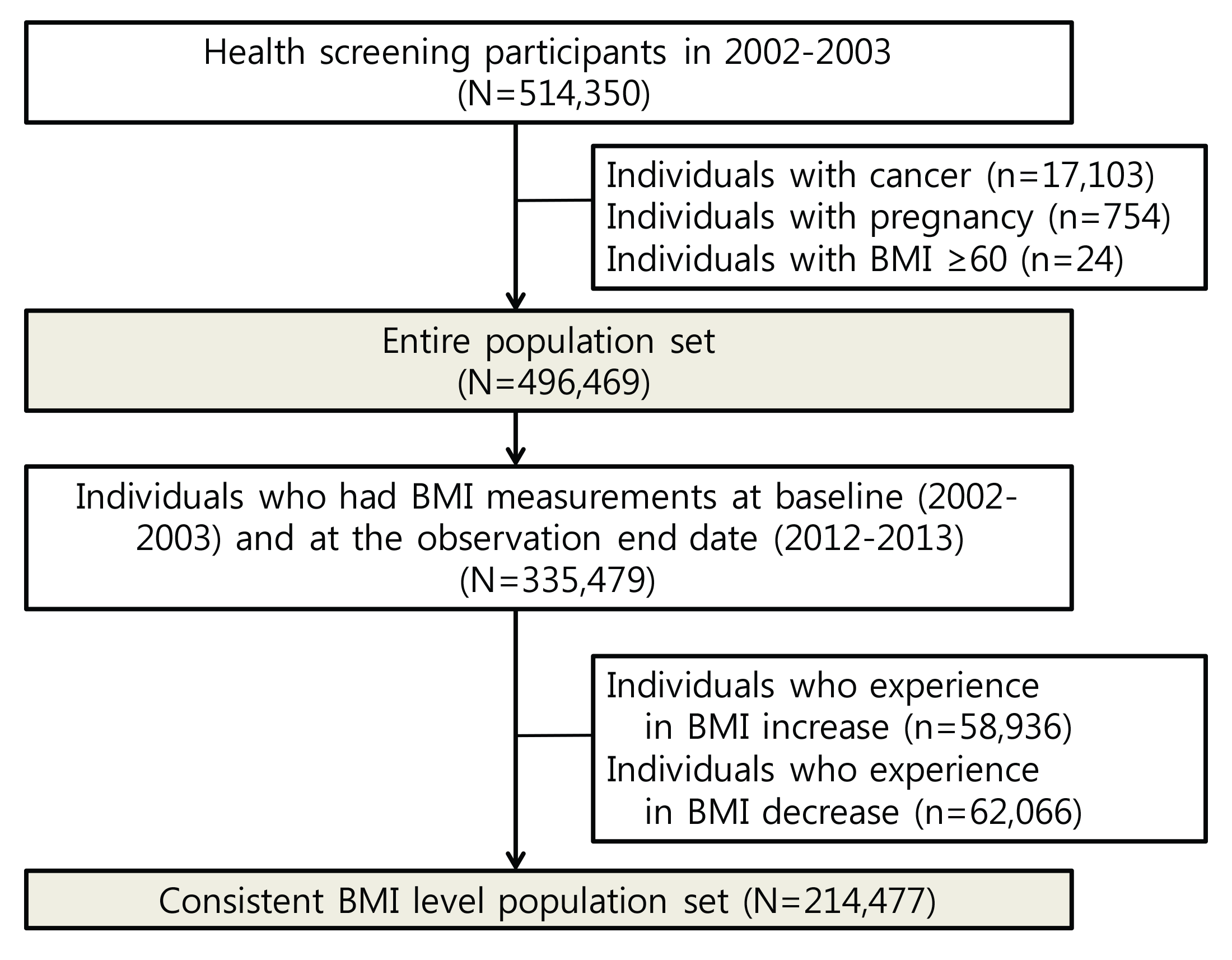

Supplement: S1 Fig — (TIF) [file pone.0197057.s007.tif]
